# 

**Published:** 1901-02-02

**Authors:** 


### NOTICE TO CORRESPONDENTS.

All MSS., Letters, Books for Review, and other matters intended for the Editor should be addressed The Editor, "The Hospital," The Hospital Building, 28 & 29 Southampton Street, Strand, W.C.

The Editor cannot undertake to return rejected MSS., even when accompanied by stamped directed envelope.

**Notice to Advertisers and Subscribers.**

All Advertisements, Orders for Copies of The Hospital, and Business Communications should be addressed to **THE MANAGER (Not to the Editor)**, The Hospital, 28 & 29 Southampton Street Strand, London, W.C.

The Cost of Subscription, post free, is as follows:—Per week, 2½d.; per quarter, 2s. 3d.; per half-year, 5s. 3d.; per annum, 10s. 6d. Foreign:—Per annum, 15s. 2d., payable, in all cases, in advance.

**NOTICE.—Vol. XXVIII. of "The Hospital" (April to September, 1900), in handsome cloth binding, is now on sale at the Office of this Journal, price, 7s. 6d. Cases for binding the Numbers contained in this Volume 1s. 6d. Index to Vol. XXVIII., 2½d., post free.**

**The Monthly Part for February is now ready. Price 6d., by post 8d.**

### SCRAPS AND GLEANINGS.

The Cardiff *Western Mail* Shilling Fund is approaching £3,000.

During 1900 the receipts of the Ipswich and East Suffolk Hospital were £3,048, whilst the expenditure was £2,826.

The total number of patients treated at the 15,643 hospitals helped by the Liverpool Hospital Sunday Fund last year was 224,886.

Professor Howden, of the University of Durham, has been appointed Examiner in Anatomy in the University of Edinburgh.

The nett proceeds of the bazaar in aid of the Lytham Cottage Hospital were £1,040, which has been paid into the hospital account.

At the annual dinner of the Sutton Cottage Hospital a cheque, value £120, was handed to the Chairman, being the amount of the working men's contributions.

It is proposed to erect a new isolation ward at the Milton Hospital, and the Local Government Board have been applied to by the Portsmouth Corporation for permission to raise a loan of £4,700.

The laundry machinery in the new laundry now being built at the Lincoln County Hospital is to be driven by electric motors. It is hoped also that it may be possible to introduce the electric light into the hospital.

The building of the new Wallasey Hospital has cost £12,000, and the furnishing cost another £4,000. At present the hospital is supplied with 40 beds, but it is contemplated to have two extensions each providing 40 beds.

A "Hospital Aid Society" has been formed in Cork to extend the work of the Hospital Saturday collection. The objects of the society are to organise collections of small sums in various districts throughout the county.

During the year 1900, 714 children were admitted to the St. Leonards Convalescent Home for Poor Children, as against 721 in 1899. Since 1880, when the number admitted was 240, the average yearly increase in the number of children admitted was 450.

The Bishop of Stepney, as Vice-President, together with the Chairman, Treasurer, and a representative of the Committee, make an urgent appeal for £34,500 for the enlargement of the North-Eastern Hospital for Children, which at present contains only 57 beds, and this accommodation has to serve a population of over 500,000.

### The Student of Medicine.

### APPOINTMENTS.

**H. Allen,** M.R.C.S., L.R.C.P. Lond., appointed District Medical Officer of the Crediton Union. **Percival E. Barber,** B.A.Camb., L.R C.P.Lond., M.R.C.S.Eng., appointed Honorary Medical Officer to the Jessop Hospital for Women, Sheffield. **T. W. Bartlett,** L.R.C.P., L.R.C.S.Edin., appointed Medical Officer for the Fourth District of the Halstead Union. **G. A Brown,** M.B., C.M.Glasg., appointed Medical Officer of Health for the Burgh of Partick. **E. Coleman,** M.R.C.S.Eng., L.R.C.P.Lond., appointed Medical Officer for the Blankney District of the Sleaford Hospital. **Sidney Davies,** M.A., M.D.Oxon, D.P.H.Camb., appointed Medical Officer of Health for the Borough of Woolwich. **Hy. Noel N. Dodd,** L.R.C.P., L.R.C.S.Edin., L.F.P.S.Glasg., appointed Assistant House and Visiting Surgeon to the Stockport Infirmary. **R. C. Field,** M.B.Lond., appointed District Medical Officer to the Patrington Union. **G. A. T. Fox,** M.R.C.S.Eng., L.R.C.P.Lond., appointed District Medical Officer to the Dore Union. **Patrick J. Hamilton,** M.B., B.Ch., B.A.O., R.U.I., appointed Medical Officer and Medical Officer of Health, Arklow Dispensary District, Rathdrum Union, co. Wicklow. **T. Holmes,** M.B.Lond., appointed Medical Officer for the South District of the Lancaster Union. **Robert Howden,** M.B., C.M.Edin., appointed Examiner in Anatomy in the University of Edinburgh. **John J. Huey,** L.S.A., appointed District Medical Officer of the Doncaster Union. **H. W. Jackson,** M.D.Lond., appointed District Medical Officer to the Middlesbrough Union. **Edward Long Jacob,** B.A.Lond., M.R.C.S.Eng., appointed Medical Officer of Health for the Reigate Urban and Rural Sanitary Districts. **T. D. McLaren,** M.B., B.S.Edin.. appointed Medical Officer for the Litcham District of the Mitford and Launditch Union. **J. T. Macnamara,** L.R.C.P.Lond., L.R.C.S.I,, appointed Medical Officer of the Ladywell Workhouse of the St. Olave's Union. **Eugene M. Niall,** M.B.Lond., appointed House Surgeon to the West London Hospital. **Wm. Fletcher Oyston,** M.B., Ch.B.Vict., appointed District Medical Officer to the Northallerton Union. **M. Parry-Jones,** M.D.Lond., appointed Honorary Physician to the Derbyshire Royal Infirmary. **Thomas Priestley,** M.R.C.S., L.R.C.P.Lond., appointed Medical Officer for the Neepsend District of the Sheffield Union. **Hugh Mallinson Rigby,** M.B., B.S.Lond., F.R.C.S.Eng., appointed Surgeon to the Poplar Hospital for Accidents. **William Robertson,** M.D.Glasg., D.P.H., F.P.S.Glasg., appointed Medical Officer of Health for the Burgh of Leith. **Montagu Rust,** L.R.C.P. and S.Edin., appointed as a Civil Medical Officer to the troops in South Africa. **R. R. Smith,** L.R.C.P., L.R.C S.Irel., appointed Medical Officer for the Everton No. 1 District of the West Derby Union. **Peter Thompson,** M.D.Vict., appointed Lecturer on Anatomy to the Middlesex Hospital Medical School. **T. W. Turner,** M.R.C.S., L.R.C.P.Lond., appointed Medical Officer for the Deddington No. 1 District of the Woodstock Union. **Hunter Urquhart Walker,** appointed Divisional Surgeon of the Police for Hackney, N.E. **W. P. Warburton,** L.R.C.P., L.R.C.S.Edin., appointed Medical Officer to the Workhouse and for the Skirlaugh District of the Skirlaugh Union. **W. A. H. Wilkinson,** M.B., Ch.B.Vict, appointed Medical Officer of Health for the Gainsborough Urban District. **Chas. W. Young,** M.R.C.S., L.R.C.P.Lond., D.P.H.Camb., appointed District Medical Officer of the Luton Union.

